# Data to calculate emissions intensity for individual beef cattle reared on pasture-based production systems

**DOI:** 10.1016/j.dib.2018.01.075

**Published:** 2018-02-02

**Authors:** G.A. McAuliffe, T. Takahashi, R.J. Orr, P. Harris, M.R.F. Lee

**Affiliations:** a Rothamsted Research, North Wyke, Okehampton, Devon EX20 2SB, UK; b University of Bristol, Langford House, Langford, Somerset BS40 5DU, UK

## Abstract

With increasing concern about environmental burdens originating from livestock production, the importance of farming system evaluation has never been greater. In order to form a basis for trade-off analysis of pasture-based cattle production systems, liveweight data from 90 Charolais × Hereford-Friesian calves were collected at a high temporal resolution at the North Wyke Farm Platform (NWFP) in Devon, UK. These data were then applied to the Intergovernmental Panel on Climate Change (IPCC) modelling framework to estimate on-farm methane emissions under three different pasture management strategies, completing a foreground dataset required to calculate emissions intensity of individual beef cattle.

## Specifications table

**Table:** 

Subject area	Agricultural sciences
More specific subject area	Livestock Science
Type of data	Figures
How data was acquired	On-farm data collection (liveweight), modelling (methane emissions)
Data format	Raw (liveweight), analysed (methane emissions)
Experimental factors	Ninety (90) Charolais × Hereford-Friesian cattle randomly allocated across treatments
Experimental features	Farm-scale grazing trial with three different pasture management strategies
Data source location	Okehampton, Devon, UK (50°46′10″N, 3°54′05″W)
Data accessibility	Within this article
Related research article	G.A. McAuliffe, T. Takahashi, R.J. Orr, P. Harris, M.R.F. Lee, Distributions of emissions intensity for individual beef cattle reared on pasture-based production systems, J. Clean. Prod. 171 (2018) 1672–1680.

## Value of the data

• Data were obtained from a farm-scale grazing trial, providing insight into economic-environmental trade-offs associated with livestock production systems on commercial farms.
• Adherence to the Intergovernmental Panel on Climate Change (IPCC) modelling framework enables international comparisons of on-farm greenhouse gas emissions.
• The resultant high-resolution dataset can be used to study the temporal variability of economic and environmental performance for individual cattle reared on pasture.

## 1 Data

**Fig. 1:**
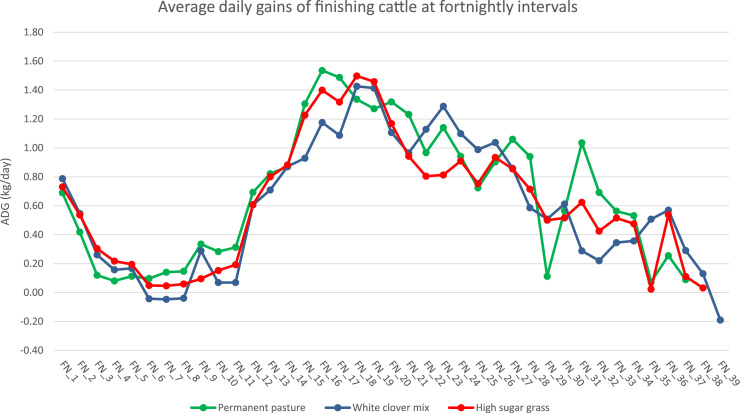
Temporal variations in average daily gains. FN = fortnight.

**Fig. 2:**
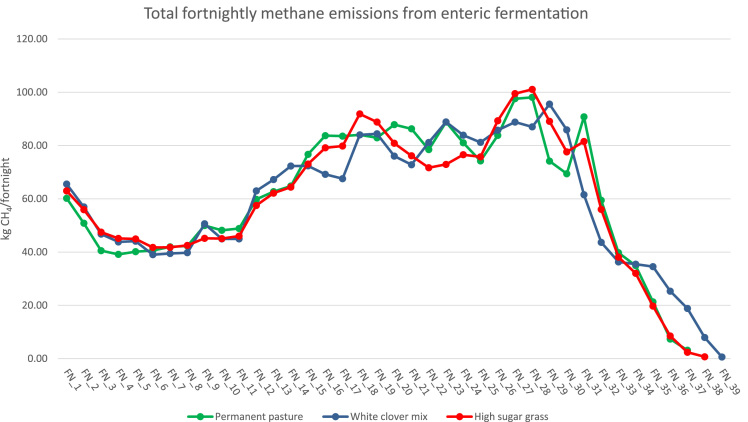
Temporal variations in methane emissions from enteric fermentation. The values are aggregated across 30 cattle under each system. FN = fortnight.

**Fig. 3:**
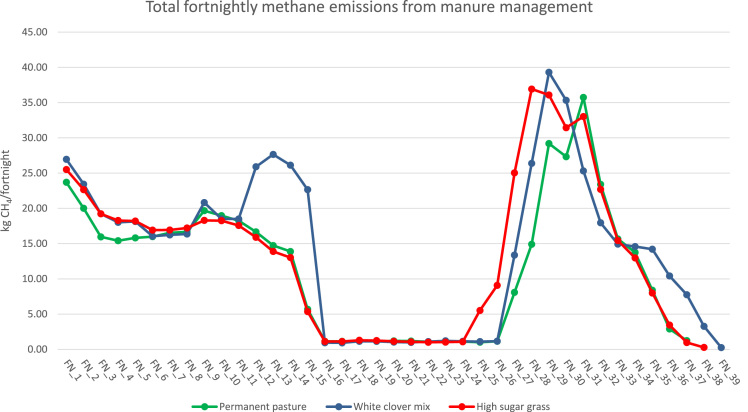
Temporal variations in methane emissions from manure management. The values are aggregated across 30 cattle under each system. FN = fortnight.

With increasing concern about environmental burdens originating from livestock production, the importance of farming system evaluation has never been greater. The data presented here were collected from the North Wyke Farm Platform (NWFP), a farm-scale grazing trial in Devon, UK, in order to study economic-environmental trade-offs associated with cattle rearing under conditions relevant to commercial production. These include: temporal variations in average daily gains (ADG) (Fig. 1), temporal variations in methane emissions from enteric fermentation (Fig. 2), and temporal variations in methane emissions from manure management (Fig. 3). All data are available as Supplementary material. Using the method of life cycle assessment (LCA), they were subsequently utilised as foreground data to calculate emissions intensity for individual cattle on the NWFP (McAuliffe et al. [1]).

## 2 Experimental design, materials, and methods

The NWFP is located in Devon, a southwest county of England, UK (50°46′10″N, 3°54′05″W) and consists of three hydrologically isolated small-scale (21 ha) livestock farms known as “farmlets”. Each of the three farmlets operates under a different pasture management system, with swards of: (1) permanent pasture, (2) white clover (*Trifolium repens*)/high sugar perennial ryegrass (*Lolium perenne*) mix, and (3) high sugar perennial ryegrass monoculture. Orr et al. [2] provide further information on the NWFP's design concept and farming operation.

Every autumn, 30 Charolais × Hereford-Friesian calves enter each farmlet at the point of weaning. At this time, animals are blocked between sexes and then randomly allocated to the farmlets from an adjacent but separate cow-calf operation, of which grasslands are permanent pasture similar to the system (1) above. After entering the NWFP, animals are typically housed from October to April to avoid destruction of soil structure during the wet season, then moved and kept outdoors on their respective pastures until they reach target weights of ca. 555 kg for heifers and 620 kg for steers and estimated meat quality scores (RPA [3]) of “R” (conformation) and “4L” (fat). If animals do not meet these finishing criteria at pasture, a second housing period may be required. Throughout housing periods, animals are fed silage comprising grasses and legumes harvested from their own allocated systems. While the NWFP's general principle is to finish cattle solely off pasture and silage, depending on the quantity and quality of silage produced in any particular year, strategic supplementary feed to balance energy and protein demands may be used and recorded. When strategic feeding occurs, its quantity is set at a uniform rate across animals to minimise confounding effects.

The present data follow 90 cattle (30 per farmlet) that were born in the spring of 2014, covering the period from October 2014, when they were weaned, to their departure to the slaughterhouse on meeting weight and carcass specification targets, around December 2015 for the majority of the animals. Throughout this study period, individual animals were weighed every two to four weeks using a cattle crush and weigh head, providing a high temporal resolution for ADG. Digestible energy values of forage (during grazing periods) and silage (during housing periods) were also quantified at the same frequency under the protocol described by McAuliffe et al. [1].

Methane emissions arising from livestock were calculated using a modified IPCC Tier 2 approach (IPCC [4]). In order to examine both temporal changes in emissions and the effects of animal heterogeneity, these values were derived for each animal for each time period (between two weighing events) using the weighing records and digestible energy information described above. Calculations were programmatically automated and linked to the NWFP database so as to apply different parameters depending on the animal's age, location and feed being consumed.

## Footnotes

Appendix A Supplementary data associated with this article can be found in the online version at doi:10.1016/j.dib.2018.01.075.
