# Supplementary material for: Data to calculate emissions intensity for individual beef cattle reared on pasture-based production systems
Source: Data Brief. 2018 Feb 2;17:570–4. doi: 10.1016/j.dib.2018.01.075 (PMC5852278; doi:10.1016/j.dib.2018.01.075)
Supplement: Supplementary file 1 — Supplementary material. [file mmc1.pdf]

Conflict of Interest Declaration

Authors declare no conflict of interest associated with this publication.
